# Empathy at the Gates: Reassessing Its Role in Moral Decision Making

**DOI:** 10.3389/fpsyg.2022.800752

**Published:** 2022-01-27

**Authors:** Afreen S. Khalid, Stephan Dickert

**Affiliations:** ^1^Department of Cognitive Psychology, Institute of Psychology, University of Klagenfurt, Klagenfurt, Austria; ^2^Department of Marketing, School of Business and Management, Queen Mary University of London, London, United Kingdom

**Keywords:** empathy, compassion, affective empathy, norms, value-based choice, rationality

## Introduction

A plethora of research conducted in the past decades has shown that empathy can be essential in guiding and motivating prosocial behavior (e.g., Batson et al., 1981; Eisenberg and Miller, 1987; Batson, 2011; Dickert et al., 2011; Erlandsson et al., 2015). However, there is still considerable debate around whether empathy-driven altruism does more harm than good. For example, several parochial biases have been linked to empathy (Bloom, 2016), such as a preference for helping in-group over out-group members (Cikara and Fiske, 2012) and identifiable over statistical victims (Small and Loewenstein, 2003; Kogut and Ritov, 2005). In response to the limited and scope-insensitive empathic responses to large numbers of victims (Slovic, 2007; Västfjäll et al., 2014), suggestions have been put forward that moral decisions should be guided by rational compassion rather than empathy (Bloom, 2016).

In this article, we attempt to clarify some main points of confusion in the discourse surrounding the merits and pitfalls of empathy. In doing so, we also lay out several possible directions for future research.

## Defining Empathy

Much of the disagreement surrounding the utility of empathy can be attributed to the non-overlapping definitions of empathy used in the field. The fuzzy definitions of empathy and compassion have also been pointed out by several different researchers (Neumann et al., 2015; Cuff et al., 2016; Västfjäll et al., 2017; Eklund and Meranius, 2021; Scheffer et al., 2021). Indeed, the lack of a consistent definition of empathy and compassion is a crucial issue which holds back progress in this area of research.

Most commonly, empathy is described as a multi-factorial construct, with (1) an affective component (experience-sharing), which involves feeling the emotions of another person, (2) a cognitive component (perspective-taking/mentalizing), which involves perceiving another person's thoughts or feelings, and (3) a motivational component (compassion/empathic concern), which involves an emotional response that creates the urge to foster the well-being of others (Batson et al., 1997; Decety and Cowell, 2014; Zaki, 2014; Marsh, 2018).

While empathy is described as having several distinct components, critics of empathy (Prinz, 2011; Singer and Klimecki, 2014; DeSteno, 2015; Bloom, 2017) often equate it *exclusively* to its affective component and consider compassion a distinct process with the capacity to motivate prosocial behavior in more effective ways than empathy (Scheffer et al., 2021). On the other hand, compassion is considered a sub-facet of empathy by many researchers (Decety and Cowell, 2014). Whether compassion should be classified as a sub-component of empathy or if it is a separate process is still an open question which future research should examine. Recent research has already begun investigating the different components of empathy and whether they are separate or co-occur in daily life experiences (for review see Weisz and Cikara, 2020; Depow et al., 2021). One possibility laid out by Decety (2021), based on an understanding of the evolutionary roots of empathy, is that empathy's core constituents are (1) emotion sharing, which evolved to facilitate cooperation, and (2) concern for others' well-being, which is an adaptive mechanism that evolved to facilitate the care of offspring. According to this multidisciplinary perspective, these core components are influenced by elements such as perspective-taking and theory of mind. While this is a promising theoretical framework, in light of the definitional challenge of empathy, more research is needed to examine what components constitute empathy, to what degree and in which contexts these components co-activate, and how these components differentially (or collectively) facilitate prosocial behavior.

## Empathy as a Value-Based Choice

Empathy is often described both as an automatic, intractable reaction and as a limited resource that can be depleted with overuse (Decety and Cowell, 2014; Bloom, 2016). These limitations of empathy have been cited as a reason behind its parochialism and insensitivity to statistical victims. However, alternative accounts of empathy have been put forward (Zaki, 2014; Cameron et al., 2017) which argue that, instead of being only intuitive and uncontrollable, empathy can be a motivated phenomenon which individuals choose to approach or avoid based on perceived costs and benefits. The malleability of empathy implies that suggestions to discard empathy as a poor moral compass may be premature because empathy can be shaped toward more positive outcomes.

In support of this view, research that looks at the *cognitive effort costs* of empathizing suggests that empathy's biases may not be inherent in empathy itself but caused by shifts in incentives. When people view empathy as hard work, they might choose to avoid it if given the opportunity (Cameron et al., 2019). People are also more willing to bear the costs of empathy when they have more incentive to do so, such as when it involves members of their kin. This is in line with previous research showing that people avoid empathy in situations where the costs of helping would be too high (Shaw et al., 1994).

One limitation of these studies is that most of them either used a broad definition of empathy or just investigated affective empathy. Whether different components of empathy are affected differentially by motivational cues is still an important question for future research (Ferguson et al., 2020). Another crucial point to note is that while critics of empathy usually recognize that empathy is subject to motivational biases, they fail to make the same judgment for compassion. However, results of a recent study support the view that compassion suffers from some of the same biases as empathy, perhaps even to a greater degree (Scheffer et al., 2021). This study indicated that participants perceived compassion as more cognitively costly than empathy, especially when applied to strangers, which is at odds with the view of compassion as less exhausting than empathy and more likely to lead to sustained helping (Bloom, 2017). Indeed, scope insensitivity has been observed for a range of emotions, including empathy, sympathy, and compassion (Kogut and Ritov, 2005; Dickert and Slovic, 2009; Cameron and Payne, 2011; Västfjäll et al., 2014).

Some of the above research conceptualizes empathy as a value-based choice. From a value-based perspective, individuals make a choice based on its relative subjective value. Thus, under this framework, people might *choose* to feel empathy by (un)consciously considering its costs (effort, time, money) and benefits (monetary rewards, norm conformity, status). Indeed, research in moral decision-making has begun to make the case for a value-based framework which can help reconcile conflicting findings in the literature (Cameron et al., 2017; Pärnamets et al., 2020).

While more work is needed to understand whether (or when) empathy is best conceptualized as a value-based choice or if it is, as often suggested, an automatic, intuitive response unaffected by value processes, empathy can be a dynamic system that shifts with changing values. Consequently, if the aim is to expand the circle of individuals for whom we feel empathy (Singer, 1981), then changing underlying motives or incentives might be a useful direction toward this.

## Affective Empathy: Not All Bad

Affective empathy is subject to a number of biases. We can be insensitive to the number of those suffering (Slovic, 2007; Västfjäll et al., 2014; Dickert et al., 2015), biased toward in-group members (Harris and Fiske, 2006), and prefer helping identifiable victims over faceless masses (Small et al., 2007; Lee and Feeley, 2016). Our internal “empathy meters” often don't scale up with the magnitude of the problem.

However, this aspect does not necessitate the dismissal of empathy as a whole. For one, empathy is an evolved mechanism that serves adaptive functions when it comes to caring for the young and coordinating toward achieving a shared goal (Preston and de Waal, 2002; Cohen et al., 2006; Preston, 2013; de Waal and Preston, 2017). It is possible that intuitive, affective empathy constitutes a primary mechanism for helping in the first place (Decety, 2021). Thus, it could very well serve as an activation process by which further cost and benefit calculations are triggered.

Moreover, some evidence suggests that empathy has expanded to a wider circle of individuals over the past few decades (Pinker, 2011). The increasing efforts toward globalization in several domains of life such as economics, culture, politics and communication as well as technological innovations may create the initial sparks of empathy that allow people to consider the perspective of those outside their immediate in-group (Bhagwati and MacMillan, 2004; Pinker, 2011). These, in-turn, may pave the ways for policies and norms protecting the rights of minority groups, which have already been embedded into the moral psychology of some cultures. Ultimately, these policies and norms may affect our experience of empathy. Indeed, this is consistent with research on how incentives and culture affect the empathic experience (Atkins et al., 2016; Nook et al., 2016). This suggests that increasing opportunities for intuitive empathy combined with changing existing norms and incentives will be a key aspect of expanding our circle of empathy. In summary, affective empathy is an instinctive, evolved phenomenon which is important for social functioning. While it would appear that it is often intuitive and automatic, the history of moral progress and evidence from contemporary research in psychology suggests that empathy may reflect values which are, at least in part, changeable.

## Conclusion: Motivated by the Heart, Guided by the Head

The research summarized here suggests several ways for empathy research to move forward. First, it is important to figure out exactly which sub-components fall under the empathy umbrella. How these sub-components operate independently and in concert is another crucial question for future research. Future research should also test whether interventions influence all components of empathy, or whether they are more effective for certain components over others. The efficacy of different motivational cues (such as financial rewards, social rewards or psychological benefits like warm glow) should also be tested on each empathic sub-component.

Further, while claims have been made by researchers on how our circle of empathy has expanded over the centuries, causal research on how our morals change is limited (Bloom, 2010; Andreoni et al., 2021). There is some research which suggests that decision framing matters for aiming to expand our circle of moral regard (Laham, 2009). However, our understanding of this process would benefit from longitudinal research on what processes trigger changes in norms and emotions of individuals and what the causal chain of this process is.

On a related note, some scholars have also argued that initiators of norm abandonment (i.e., trendsetters) are a crucial part of the norm change process (Bicchieri and Funcke, 2018). Effective Altruism (EA)–a movement based on using evidence and reason to do the most good–attempts to initiate such a change (Caviola et al., 2021). The central message espoused by its proponents is that individuals in affluent countries are morally obligated to donate to socially distant individuals living in extreme poverty (Singer, 1972, 2015). Focusing on aspects such as effectiveness and efficacy also allows comparisons and makes help more quantifiable. Recent research suggests that donors do not instinctively consider the efficacy of their donations (Burum et al., 2020). Making efficacy salient could shift moral norms and hence make people more sensitive to it.

While the emphasis on effectiveness, efficacy, and rationality may seem like a blow for empathy, this need not be the case. Affective processes might be necessary to create the initial spark that lights the fire of moral progress, as affect-rich stimuli often motivate prosocial behavior (Small and Loewenstein, 2003; Kogut and Ritov, 2005; Erlandsson et al., 2015; Dickert et al., 2016). While empathy and/or compassion are the fuel that kick starts our morality, tools such as logic, critical thinking, utilitarian cost-benefit analyses, argumentation with others and reasoning based on empiricism can serve as the steering wheel–allowing us to recognize and reach our preferred destination (Decety, 2021; Pinker, 2021) and perhaps shift our very experience of empathy.

## Author Contributions

AK and SD contributed to writing and editing the manuscript. Both authors contributed to the article and approved the submitted version.

## Conflict of Interest

The authors declare that the research was conducted in the absence of any commercial or financial relationships that could be construed as a potential conflict of interest.

## Publisher's Note

All claims expressed in this article are solely those of the authors and do not necessarily represent those of their affiliated organizations, or those of the publisher, the editors and the reviewers. Any product that may be evaluated in this article, or claim that may be made by its manufacturer, is not guaranteed or endorsed by the publisher.
